# Adjunct use of radiofrequency coblation for osteochondritis dissecans in children: A case report

**DOI:** 10.1097/MD.0000000000021437

**Published:** 2020-08-28

**Authors:** Reed Estes

**Affiliations:** OrthoSports Associates, Building 3, Suite 403, Birmingham, AL.

**Keywords:** cartilage, case report, coblation, osteochondritis dissecans, radiofrequency

## Abstract

**Rationale:**

Osteochondritis dissecans (OCD) lesions involve disruption of the osteochondral unit along articular surfaces, with significant potential for joint deterioration if not managed appropriately.

**Patient concerns:**

A 15-year-old male presented with persistent and insidious right knee pain, which had worsened following a collision with another player during a basketball game, resulting in episodes of locking.

**Diagnoses:**

Magnetic resonance imaging revealed a lateral trochlear OCD extending into the anterior lateral femoral condyle.

**Interventions:**

Chondral fraying was observed along the margins of the OCD. Retrograde drilling ensued with use of a 0.045-inch Kirschner wire throughout the lesion to a depth that would allow for penetration of healthy underlying subchondral bone to create an influx of healing factors. Three resorbable pegs were arthroscopically placed through an accessory portal overlying the lesion to stabilize the fracture and compress the gapped cartilage mantle to reduce flow of synovial fluid behind the lesion. Bipolar radiofrequency coblation was used to stabilize the chondral fraying and seal the gap along the periphery of the lesion.

**Outcomes:**

The patient was put on a nonweight bearing protocol for 6 weeks, after which crutches and brace were discontinued, but therapy persisted. Repeat imaging at 3 months demonstrated excellent interval healing. The patient was released to slowly engage impact activities. Although he returned at approximately 8 months postoperatively with a contralateral anterior cruciate ligament tear, he reported the operative knee with the OCD was doing extremely well.

**Lessons:**

Radiofrequency coblation appears to be a viable strategy as an adjunct to management for OCD in children.

## 1 Introduction

Osteochondritis dissecans (OCD) lesions involve a disruption in the underlying subchondral bone, leading to potential overlying cartilage injury even though initial symptoms may be minimal. Multiple etiologies have been proposed for OCD lesions, including both biological (eg, genetic causes, underlying endocrine abnormalities) and mechanical (eg, injury/overuse possibly related to sports at a young age).^[1]^

The incidence of OCD lesions appears to be increasing, and is highest in males and those between the ages of 11 and 15 years.^[2]^ The lateral portion of the medial femoral condyle is most common site of occurrence, representing up to 75% of these lesions.^[3–5]^ Lesions in the lateral femoral condyle are an independent risk factor for failure and patellar lesions demonstrate an increased risk for persistent knee pain.^[6,7]^

This paper describes management options for OCD lesions, highlighting a case using radiofrequency coblation as an adjunct for cartilage debridement and stabilization. To the best of the author's knowledge, this is the first published description of using this technology in a pediatric patient with OCD lesions.

## 2 Case report

For the purposes of the following case report, approval was not required by the Institutional Review Board. Informed consent was obtained from the patient's parent for the purpose of educational and research purposes and the report was created with no identifying information.

A 15-year-old African American male was seen in consultation from a primary care sports medicine physician for evaluation of persistent and worsening right knee pain. The pain had been present for several months and began insidiously, although worsened following a collision with another player during a basketball game, prompting episodes of locking.

Examination revealed an antalgic gait. Patellofemoral exam was noted to be stable, yet he did have pain overlying the lateral trochlea with full flexion. Effusion was not observed and pain was further exacerbated with patellar grind test. Further ligamentous and special testing for the knee was negative.

**Figure 1:**
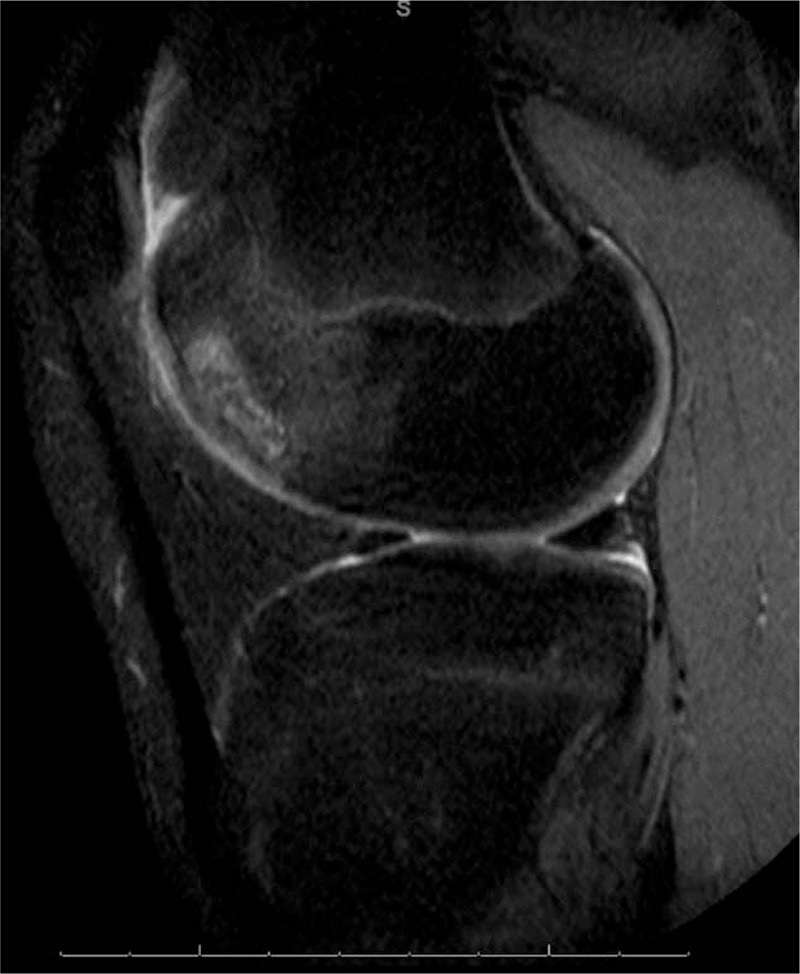
Sagittal magnetic resonance imaging demonstrating lateral trochlear osteochondritis dissecans lesion.

**Figure 2:**
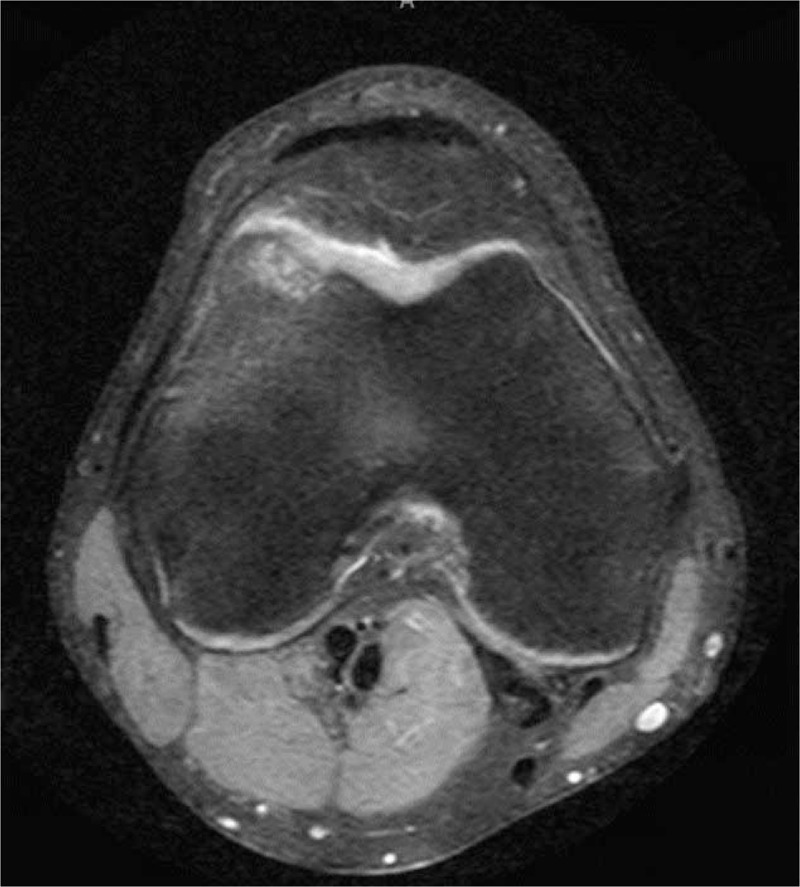
Axial magnetic resonance imaging demonstrating lateral trochlear osteochondritis dissecans lesion.

Routine anterior-posterior, lateral, Merchant and tunnel view radiographs revealed irregularity along the lateral trochlea. Secondary to the acute exacerbation and mechanical symptoms, magnetic resonance imaging was ordered. This revealed a lateral trochlear OCD extending into the anterior lateral femoral condyle (Figs. 1 and 2). Based upon these findings, he was referred to our office for evaluation.

Discussion with the family centered around options to include conservative management with activity modification, bracing, and rehabilitation, vs that of surgical intervention. Surgery was chosen based upon the duration of symptoms, presence of mechanical symptoms, and the inferior outcomes with trochlear lesions.

**Figure 3:**
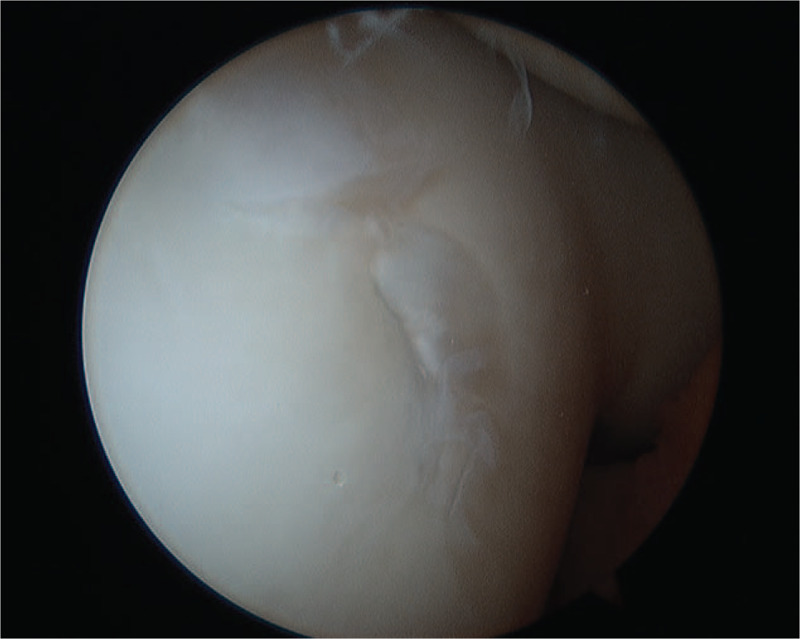
Arthroscopic imaging of osteochondritis dissecans lesion.

**Figure 4:**
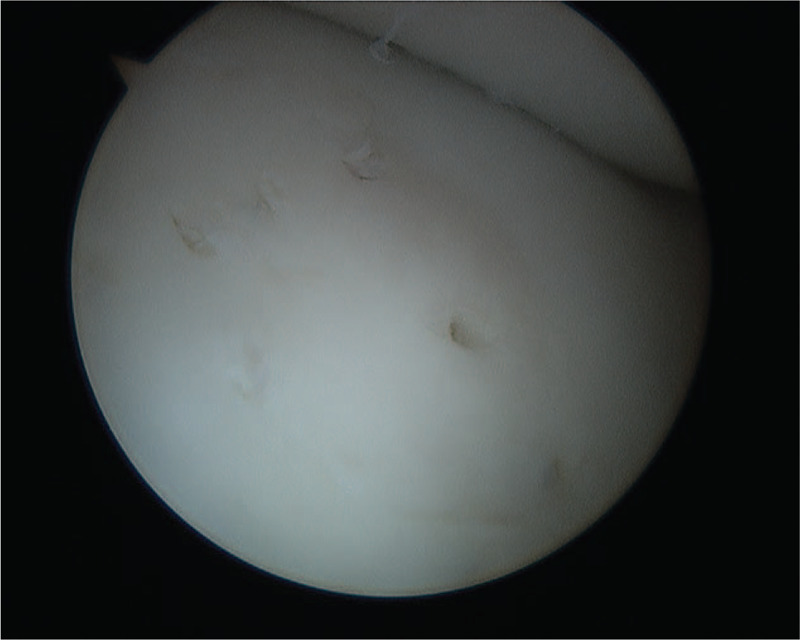
Arthroscopic imaging of osteochondritis dissecans lesion postdrilling and radiofrequency coblation.

Standard anterolateral and anteromedial arthroscopic portals were used to perform diagnostic arthroscopy of the knee. Visualization revealed the joint structures to be intact with the exception of a lateral trochlear OCD lesion, noted to be unstable to probing. Chondral fraying was observed along the margins of the OCD and the lesion was partly mobile within the donor site (Fig. 3). Retrograde drilling ensued with use of a 0.045-inch Kirschner wire along the centrum and periphery of the lesion to a depth that would allow for penetration of healthy underlying subchondral bone to create an influx of healing factors. Three resorbable pegs (LactoNail; Biomet) were arthroscopically placed through an accessory portal overlying the lesion. These served to stabilize the fracture and compress the gapped cartilage mantle to reduce flow of synovial fluid behind the lesion. Persistent chondral fraying along the edges of the lesion prompted additional need for chondroplasty and bipolar radiofrequency coblation (Werewolf Flow 50 wand; Smith and Nephew) was used to stabilize the chondral fraying and seal the gap along the periphery of the lesion (Fig. 4). The use of coblation was felt to be a crucial component of the treatment, improving stability and contour of the residual cartilage, thereby allowing for recovery of appropriate joint mechanics.

Postoperative instructions included a period of nonweight bearing for 6 weeks while wearing a hinged knee brace to allow for gradual progression of range of motion with supervised rehabilitation. Crutches and brace were discontinued at 6-week follow-up, but therapy persisted.

At approximately 3 months postoperatively, repeat imaging demonstrated excellent interval healing and the patient was released to slowly engage impact activities. Unfortunately, he was seen at approximately 8 months postoperatively with a contralateral anterior cruciate ligament tear, but reported the operative knee with the OCD was doing extremely well.

## 3 Discussion

Nonoperative strategies for OCD lesions, such as strenuous activity restriction,^[8]^ can be beneficial, especially in skeletally immature patients with low-grade lesions.^[9,10]^ However, long-term outcomes demonstrate a more complicated picture, with 32% of patients in a retrospective study having moderate-to-severe arthritis at a mean of 34 years follow-up.^[11]^

In appropriate patients, surgical interventions for OCD lesions have shown success. Internal fixation of OCD lesions in both skeletally mature and immature patients exhibits similar rates of healing and improvements in functional outcomes.^[6]^ Good long-term outcomes have been noted with biologic fixation with osteochondral plugs.^[12]^ Osteochondral allograft replacement may be an option as a salvage procedure in the setting of a loose body or unusable fragment, demonstrating reduction in pain levels and potential long-term survival of the grafts.^[13]^ A systematic review of 25 studies including 470 patients under the age of 18 years demonstrated healing regardless of technique in the majority.^[14]^

When managing cartilage damage, an ideal method should achieve a smooth surface through removal of flaps of tissue, with minimal overall “tissue effect,” defined as the depth of the removed tissue combined with that of damage to the surrounding tissue.^[15]^ This allows for preservation of joint mobility, shock absorption, and reduction of joint contact loads.

In regards to OCD lesions, stability of the overlying cartilage and regeneration of the underlying subchondral bone are paramount. Regeneration of the subchondral bone may be achieved through antegrade and retrograde drilling, or through grafting or replacement in some settings. Equally important, the cartilage stability may be improved as a result of taming the subchondral bone, but also directly through chondroplasty with debridement of flaps and irregularities at the surface to improve joint mechanics.

Options for chondroplasty include debridement with use of mechanized shavers or radiofrequency. Although both confer the advantage of arthroscopic access, they differ in regards to the ability to achieve a desirable surface smoothing and in the potential for iatrogenic cartilage injury.

Mechanical debridement with use of a shaver is disadvantageous in that it functions through gross tearing of tissue. In order to remove an appropriate amount of tissue, multiple passes may be necessary, each of which may lend to further unintended injury the neighboring tissue.^[16,17]^ Radiofrequency requires fewer passes to achieve a desirable smoothing,^[15]^ and has been noted to preserve chondrocyte viability.^[18]^

Radiofrequency involves energy passed from one electrode to a ground. In a monopolar device, the energy must be conveyed to a grounding pad, rendering temperature control more difficult. Bipolar devices pass energy between an active and ground electrode within the tip of the device, creating a more controlled local temperature. With coblation technology, as energy is passed through the tip of the device, the irrigation solution is ionized creating a plasma field, which results in localized energy secondary to molecular friction.^[19]^ Subsequent collagen denaturation occurs, followed by formation of collagen bonding as energy is removed. These bonds have been shown to have an orientation more parallel to that of the articular surface. This surface has been termed a “neo-surface,” which is proposed to confer mechanical benefit.^[20,21]^ This effect may decrease cartilage permeability, which can preserve water content and help to maintain cartilage stiffness.^[22]^

While early radiofrequency devices had significant issues with temperature control, enhanced safety features such as automated outflow control may facilitate a safer resection of tissue, minimizing unintended damage as a result of overheating. Of note, arthritic cartilage seems to be more susceptible to thermal injury than nonarthritic cartilage when tested in vitro.^[23,24]^ This may be useful in consideration of debridement of diseased tissue, in that the potential for iatrogenic damage to surrounding intact tissue may be lessened. The coblation technology used in this case aids regulation of saline outflow, decreasing the risk of harmful temperature increase within the joint.

Regardless of method chosen for chondral debridement, resection of diseased tissue comes at the price of damage to the surrounding intact tissue. Despite a greater depth of cell death with radiofrequency as compared to mechanical shaver, an important point is that the overall tissue effect is decreased with use of radiofrequency.^[15]^ In order to minimize damage to surrounding tissues, a “paintbrush” technique has been advocated to eliminate direct contact passes.^[16,19]^ By limiting direct contact with tissues, the risk of unwanted tissue damage may be decreased, as evidence demonstrates significant decline in local heat with increasing distance of the probe to tissue.^[20,25]^

Further factors that increase safety of radiofrequency include control of irrigation solution temperature as well as maintaining continuous fluid flow. It is crucial to identify fluid flow issues, particularly with the potential for a larger piece of tissue to clog the suction. With flow cessation, temperatures can rise rapidly to 80°C.^[26]^ The device used in this report uses technology to regulate outflow of saline, thereby decreasing risk of significant temperature increase.

In addition to beneficial surface smoothing, radiofrequency may have the ability to aid cartilage regeneration through the upregulation of IL-6 and IL-8.^[27]^ Although elevated temperatures may cause chondrocyte death, temperatures below 50°C to 55°C but higher than 37°C may stimulate chondrocyte metabolism.^[28]^

An important consideration, particularly in the youth population, is that of effects on the underlying subchondral bone with use of radiofrequency. When used as an adjunct to mechanical debridement for articular cartilage and meniscus, neither monopolar radiofrequency^[29]^ or bipolar radiofrequency^[30]^ have been shown to increase the incidence of osteonecrosis.

Multiple clinical studies have evaluated the effectiveness of radiofrequency in regards to outcomes and healing.^[21,31–35]^ At 1, 4, and 10 years, Spahn et al evaluated patients with concomitant chondroplasty and meniscectomy. Knee injury and Osteoarthritis Outcome Score scores were consistently higher at each time interval for the radiofrequency group. Joint space narrowing was noted in both groups, yet occurred more rapidly in the mechanical debridement group, and the rate of revision was higher in the mechanical group as well.^[31–33]^ In an in vivo second-look arthroscopy study, Voloshin et al noted greater than 50% complete or partial healing of partial thickness chondral lesions.^[34]^ While patellar lesions remain some of the most difficult to manage, improved functional scores were observed at 12 and 24 months when using radiofrequency as opposed to mechanical debridement for grade 2 and 3 lesions.^[21]^ Systematic review of 6 studies revealed improved clinical outcomes with use of bipolar radiofrequency vs mechanical debridement.^[35]^

In summary, radiofrequency coblation appears to be a viable strategy as an adjunct to management for OCD in children, although further follow up will help determine this. This surgical intervention may be considered in suitable patients who are not candidates for nonoperative management.

## Acknowledgments

The author would like to thank Smith and Nephew for background technical information related to coblation technology and financial assistance for preparation of the manuscript.

## Author contributions

**Conceptualization:** Reed Estes.

**Writing – original draft:** Reed Estes.

**Writing – review & editing:** Reed Estes.

## References

[1] Andriolo L Crawford DC Reale D Osteochondritis dissecans of the knee: etiology and pathogenetic mechanisms. a systematic review. Cartilage 2018;11:273 90. 29998741 10.1177/1947603518786557 PMC7298596

[2] Pareek A Sanders TL Wu IT Incidence of symptomatic osteochondritis dissecans lesions of the knee: a population-based study in Olmsted County. Osteoarthritis Cartilage 2017;25:1663 71. 28711583 10.1016/j.joca.2017.07.005 PMC5798004

[3] Garrett JC Osteochondritis dissecans. Clin Sports Med 1991;10:569 93. 1868560

[4] Peters TA McLean ID Osteochondritis dissecans of the patellofemoral joint. Am J Sports Med 2000;28:63 7. 10653545 10.1177/03635465000280012201

[5] Kessler JI Nikizad H Shea KG The demographics and epidemiology of osteochondritis dissecans of the knee in children and adolescents. Am J Sports Med 2014;42:320 6. 24272456 10.1177/0363546513510390

[6] Wu IT Custers RJH Desai VS Internal fixation of unstable osteochondritis dissecans: do open growth plates improve healing rate? Am J Sports Med 2018;46:2394 401. 29995442 10.1177/0363546518783737

[7] Hevesi M Sanders TL Pareek A Osteochondritis dissecans in the knee of skeletally immature patients: rates of persistent pain, osteoarthritis, and arthroplasty at mean 14-years’ follow-up. Cartilage 2018;11:291 9. 29998745 10.1177/1947603518786545 PMC7298597

[8] Andriolo L Candrian C Papio T Osteochondritis dissecans of the knee - conservative treatment strategies: a systematic review. Cartilage 2019;10:267 77. 29468901 10.1177/1947603518758435 PMC6585290

[9] Ananthaharan A Randsborg PH Epidemiology and patient-reported outcome after juvenile osteochondritis dissecans in the knee. Knee 2018;25:595 601. 29793821 10.1016/j.knee.2018.02.005

[10] Yang JS Bogunovic L Wright RW Nonoperative treatment of osteochondritis dissecans of the knee. Clin Sports Med 2014;33:295 304. 24698044 10.1016/j.csm.2013.11.003

[11] Twyman RS Desai K Aichroth PM Osteochondritis dissecans of the knee. A long-term study. J Bone Joint Surg Br 1991;73:461 4. 1670450 10.1302/0301-620X.73B3.1670450

[12] Chadli L Steltzlen C Beaufils P Neither significant osteoarthritic changes nor deteriorating subjective outcomes occur after hybrid fixation of osteochondritis dissecans in the young adult. Knee Surg Sports Traumatol Arthrosc 2019;27:740 4. 29916011 10.1007/s00167-018-5025-0

[13] Sadr KN Pulido PA McCauley JC Osteochondral allograft transplantation in patients with osteochondritis dissecans of the knee. Am J Sports Med 2016;44:2870 5. 27496906 10.1177/0363546516657526

[14] Abouassaly M Peterson D Salci L Surgical management of osteochondritis dissecans of the knee in the paediatric population: a systematic review addressing surgical techniques. Knee Surg Sports Traumatol Arthrosc 2014;22:1216 24. 23680989 10.1007/s00167-013-2531-y

[15] Lotto ML Wright EJ Appleby D Ex vivo comparison of mechanical versus thermal chondroplasty: assessment of tissue effect at the surgical endpoint. Arthroscopy 2008;24:410 5. 18375272 10.1016/j.arthro.2007.09.018

[16] Kosy JD Schranz PJ Toms AD The use of radiofrequency energy for arthroscopic chondroplasty in the knee. Arthroscopy 2011;27:695 703. 21663725 10.1016/j.arthro.2010.11.058

[17] Amiel D Ball ST Tasto JP Chondrocyte viability and metabolic activity after treatment of bovine articular cartilage with bipolar radiofrequency: an in vitro study. Arthroscopy 2004;20:503 10. 15122140 10.1016/j.arthro.2004.03.018

[18] Kaplan L Uribe JW The acute effects of radiofrequency energy in articular cartilage: an in vitro study. Arthroscopy 2000;16:2 5. 10627335 10.1016/s0749-8063(00)90119-1

[19] Wienecke H Lobenhoffer P Basic principles of radiosurgical systems and their applications in arthroscopy. Unfallchirurg 2003;106:2 12. 12552387 10.1007/s00113-002-0559-4

[20] Owens BD Stickles BJ Busconi BD Radiofrequency energy: applications and basic science. Am J Orthop (Belle Mead NJ) 2003;32:117 20. discussion 120-121. 12647875

[21] Owens BD Stickles BJ Balikian P Prospective analysis of radiofrequency versus mechanical debridement of isolated patellar chondral lesions. Arthroscopy 2002;18:151 5. 11830808 10.1053/jars.2002.29906

[22] Uthamanthil RK Edwards RB Lu Y In vivo study on the short-term effect of radiofrequency energy on chondromalacic patellar cartilage and its correlation with calcified cartilage pathology in an equine model. J Orthop Res 2006;24:716 24. 16514662 10.1002/jor.20108

[23] Kaplan LD Chu CR Bradley JP Recovery of chondrocyte metabolic activity after thermal exposure. Am J Sports Med 2003;31:392 8. 12750132 10.1177/03635465030310031101

[24] Kaplan LD Ionescu D Ernsthausen JM Temperature requirements for altering the morphology of osteoarthritic and nonarthritic articular cartilage: in vitro thermal alteration of articular cartilage. Am J Sports Med 2004;32:688 92. 15090386 10.1177/0363546503258858

[25] Kaplan LD Ernsthausen JM Bradley JP The thermal field of radiofrequency probes at chondroplasty settings. Arthroscopy 2003;19:632 40. 12861202 10.1016/s0749-8063(03)00128-2

[26] Zoric BB Horn N Braun S Factors influencing intra-articular fluid temperature profiles with radiofrequency ablation. J Bone Joint Surg Am 2009;91:2448 54. 19797581 10.2106/JBJS.H.01552

[27] Enochson L Sönnergren HH Mandalia VI Bipolar radiofrequency plasma ablation induces proliferation and alters cytokine expression in human articular cartilage chondrocytes. Arthroscopy 2012;28:1275 82. 22480788 10.1016/j.arthro.2012.01.005

[28] Benton HP Cheng TC MacDonald MH Use of adverse conditions to stimulate a cellular stress response by equine articular chondrocytes. Am J Vet Res 1996;57:860 5. 8725814

[29] Turker M Çetik Ö Çirpar M Postarthroscopy osteonecrosis of the knee. Knee Surg Sports Traumatol Arthrosc 2015;23:246 50. 23443330 10.1007/s00167-013-2450-y

[30] Cetik O Cift H Comert B Risk of osteonecrosis of the femoral condyle after arthroscopic chondroplasty using radiofrequency: a prospective clinical series. Knee Surg Sports Traumatol Arthrosc 2009;17:24 9. 18758748 10.1007/s00167-008-0604-0

[31] Spahn G Kahl E Mückley T Arthroscopic knee chondroplasty using a bipolar radiofrequency-based device compared to mechanical shaver: results of a prospective, randomized, controlled study. Knee Surg Sports Traumatol Arthrosc 2008;16:565 73. 18327566 10.1007/s00167-008-0506-1

[32] Spahn G Klinger HM Mückley T Four-year results from a randomized controlled study of knee chondroplasty with concomitant medial meniscectomy: mechanical debridement versus radiofrequency chondroplasty. Arthroscopy 2010;26: 9 Suppl: S73 80. 20810095 10.1016/j.arthro.2010.02.030

[33] Spahn G Hofmann GO von Engelhardt LV Mechanical debridement versus radiofrequency in knee chondroplasty with concomitant medial meniscectomy: 10-year results from a randomized controlled study. Knee Surg Sports Traumatol Arthrosc 2016;24:1560 8. 26429567 10.1007/s00167-015-3810-6

[34] Voloshin I Morse KR Allred CD Arthroscopic evaluation of radiofrequency chondroplasty of the knee. Am J Sports Med 2007;35:1702 7. 17644661 10.1177/0363546507304328

[35] Rocco P Lorenzo DB Guglielmo T Radiofrequency energy in the arthroscopic treatment of knee chondral lesions: a systematic review. Br Med Bull 2016;117:149 56. 26862117 10.1093/bmb/ldw004

